# Environmental DNA analysis as an emerging non-destructive method for plant biodiversity monitoring: a review

**DOI:** 10.1093/aobpla/plac031

**Published:** 2022-07-02

**Authors:** Pritam Banerjee, Kathryn A Stewart, Gobinda Dey, Caterina M Antognazza, Raju Kumar Sharma, Jyoti Prakash Maity, Santanu Saha, Hideyuki Doi, Natasha de Vere, Michael W Y Chan, Pin-Yun Lin, Hung-Chun Chao, Chien-Yen Chen

**Affiliations:** Department of Biomedical Sciences, Graduate Institute of Molecular Biology, National Chung Cheng University, 168 University Road, Min-Hsiung, Chiayi County 62102, Taiwan; Department of Earth and Environmental Sciences, National Chung Cheng University, 168 University Road, Min-Hsiung, Chiayi County 62102, Taiwan; Institute of Environmental Science, Leiden University, 2333 CC Leiden, The Netherlands; Department of Biomedical Sciences, Graduate Institute of Molecular Biology, National Chung Cheng University, 168 University Road, Min-Hsiung, Chiayi County 62102, Taiwan; Department of Earth and Environmental Sciences, National Chung Cheng University, 168 University Road, Min-Hsiung, Chiayi County 62102, Taiwan; Department of Theoretical and Applied Science, University of Insubria, Via J.H. Dunant, 3, 21100 Varese, Italy; Department of Earth and Environmental Sciences, National Chung Cheng University, 168 University Road, Min-Hsiung, Chiayi County 62102, Taiwan; Department of Chemistry and Biochemistry, National Chung Cheng University, 168 University Road, Min-Hsiung, Chiayi County 62102, Taiwan; Department of Chemistry, School of Applied Sciences, KIIT Deemed to be University, Bhubaneswar, Odisha 751024, India; Post Graduate Department of Botany, Bidhannagar College, Salt Lake City, Kolkata 700064, India; Graduate School of Information Science, University of Hyogo, 7-1-28 Minatojima-minamimachi, Chuo-ku, Kobe 650-0047, Japan; Natural History Museum of Denmark, University of Copenhagen, Øster Voldgade 5-7, 1350 Copenhagen K; Department of Biomedical Sciences, Graduate Institute of Molecular Biology, National Chung Cheng University, 168 University Road, Min-Hsiung, Chiayi County 62102, Taiwan; Department of Earth and Environmental Sciences, National Chung Cheng University, 168 University Road, Min-Hsiung, Chiayi County 62102, Taiwan; Department of Chemistry and Biochemistry, National Chung Cheng University, 168 University Road, Min-Hsiung, Chiayi County 62102, Taiwan; Department of Earth and Environmental Sciences, National Chung Cheng University, 168 University Road, Min-Hsiung, Chiayi County 62102, Taiwan; Department of Earth and Environmental Sciences, National Chung Cheng University, 168 University Road, Min-Hsiung, Chiayi County 62102, Taiwan; Center for Nano Bio-Detection, Center for Innovative Research on Aging Society, AIM-HI, National Chung Cheng University, Chiayi 62102, Taiwan

**Keywords:** DNA barcoding, DNA metabarcoding, environmental DNA (eDNA), molecular ecology, non-destructive biodiversity monitoring, plant conservation, population management

## Abstract

Environmental DNA (eDNA) analysis has recently transformed and modernized biodiversity monitoring. The accurate detection, and to some extent quantification, of organisms (individuals/populations/communities) in environmental samples is galvanizing eDNA as a successful cost and time-efficient biomonitoring technique. Currently, eDNA’s application to plants remains more limited in implementation and scope compared to animals and microorganisms. This review evaluates the development of eDNA-based methods for (vascular) plants, comparing its performance and power of detection with that of traditional methods, to critically evaluate and advise best-practices needed to innovate plant biomonitoring. Recent advancements, standardization and field applications of eDNA-based methods have provided enough scope to utilize it in conservation biology for numerous organisms. Despite our review demonstrating only 13% of all eDNA studies focus on plant taxa to date, eDNA has considerable environmental DNA has considerable potential for plants, where successful detection of invasive, endangered and rare species, and community-level interpretations have provided proof-of-concept. Monitoring methods using eDNA were found to be equal or more effective than traditional methods; however, species detection increased when both methods were coupled. Additionally, eDNA methods were found to be effective in studying species interactions, community dynamics and even effects of anthropogenic pressure. Currently, elimination of potential obstacles (e.g. lack of relevant DNA reference libraries for plants) and the development of user-friendly protocols would greatly contribute to comprehensive eDNA-based plant monitoring programs. This is particularly needed in the data-depauperate tropics and for some plant groups (e.g., Bryophytes and Pteridophytes). We further advocate to coupling traditional methods with eDNA approaches, as the former is often cheaper and methodologically more straightforward, while the latter offers non-destructive approaches with increased discrimination ability. Furthermore, to make a global platform for eDNA, governmental and academic-industrial collaborations are essential to make eDNA surveys a broadly adopted and implemented, rapid, cost-effective and non-invasive plant monitoring approach.

## Teaser

Environmental DNA (eDNA) analysis has recently become a successful cost and time-efficient biomonitoring technique. Application of eDNA also has considerable potential for plants, for the successful detection of invasive, endangered and rare species, and community-level interpretations. Monitoring methods using eDNA were found to be equal to or more effective than traditional methods; however, species detection increased when the two methods were combined. Currently, elimination of potential obstacles and the development of user-friendly protocols would greatly contribute to comprehensive eDNA-based plant monitoring programs. This is particularly needed in the data-depauperate tropics and for some less-concern plant groups.

## Introduction

The deterioration of biodiversity is accelerating at an unprecedented rate (Arneth *et al.* 2020), with 25 % of all monitored populations (Bongaarts 2019), and a staggering 39 % of vascular plants in particular (Antonelli *et al.* 2020; Nic Lughadha *et al.* 2020) currently threatened with extinction, forewarning a phase of global mass extinction (Myers 1990). In fact, plant diversity underpins all ecosystem functioning, suggesting that plant community loss will likely accelerate other biodiversity declines (Cardinale *et al.* 2012; Wang *et al.* 2020), and further impact the various ecosystem services that humans rely upon (Turnbull *et al.* 2016). Without strong conservation strategies and implementation, biodiversity integrity could reach a limit of destabilization, thereby reducing the Earth’s ability to resist abrupt change (viz. anthropogenic perturbations; Arneth *et al.* 2020). However, conservation efforts directed towards plant diversity can be hampered by a lack of monitoring data required for prioritizing conservation action, representing often diffuse, difficult to access, or outdated information, ultimately resulting in poorly designed management schemes (Corlett 2016). Thus, to prevent further loss of biodiversity, we need to innovate, modernize and prioritize plant conservation and management monitoring programs.

In traditional monitoring systems across taxa, organisms are detected by visual and/or acoustic identification, or through manual collection methods. All of these require the help of taxonomic experts; a commodity in rapid decline (Jorgensen *et al.* 2020). Assuming that experts can be utilized, there still remains high sampling/analysis costs (Qu and Stewart 2019), the risk of misidentification, incorrect detection due to phenotypic plasticity, failure to identify cryptic species and potentially incorrect differentiation of individuals in juvenile stages (Eiler *et al.* 2018). It is also nearly impossible to detect all the members of a particular community simultaneously, thus making ecosystem-level inferences difficult or reliant on taxonomic proxies (Eiler *et al.* 2018). Additionally, collection methods further risk injury to both organisms and researchers—an important consideration especially for rare organisms at low density, or places where sampling is difficult. Perhaps, most importantly, individuals of threatened taxa are often discouraged or even banned from collection regimes. In conclusion, relying solely on traditional monitoring methods can be more time-consuming, costly, potentially invasive/destructive and inaccurate, making conservation efforts unsuccessful even for species of ecological concern (Thomsen and Willerslev 2015; Piggott *et al.* 2021). Therefore, alternative methods (coupled or stand-alone) need to be considered for fast, cost-effective and large-scale plant biodiversity monitoring (Deiner *et al.* 2021): an especially pressing ecological and political issue.

**Figure 1.**
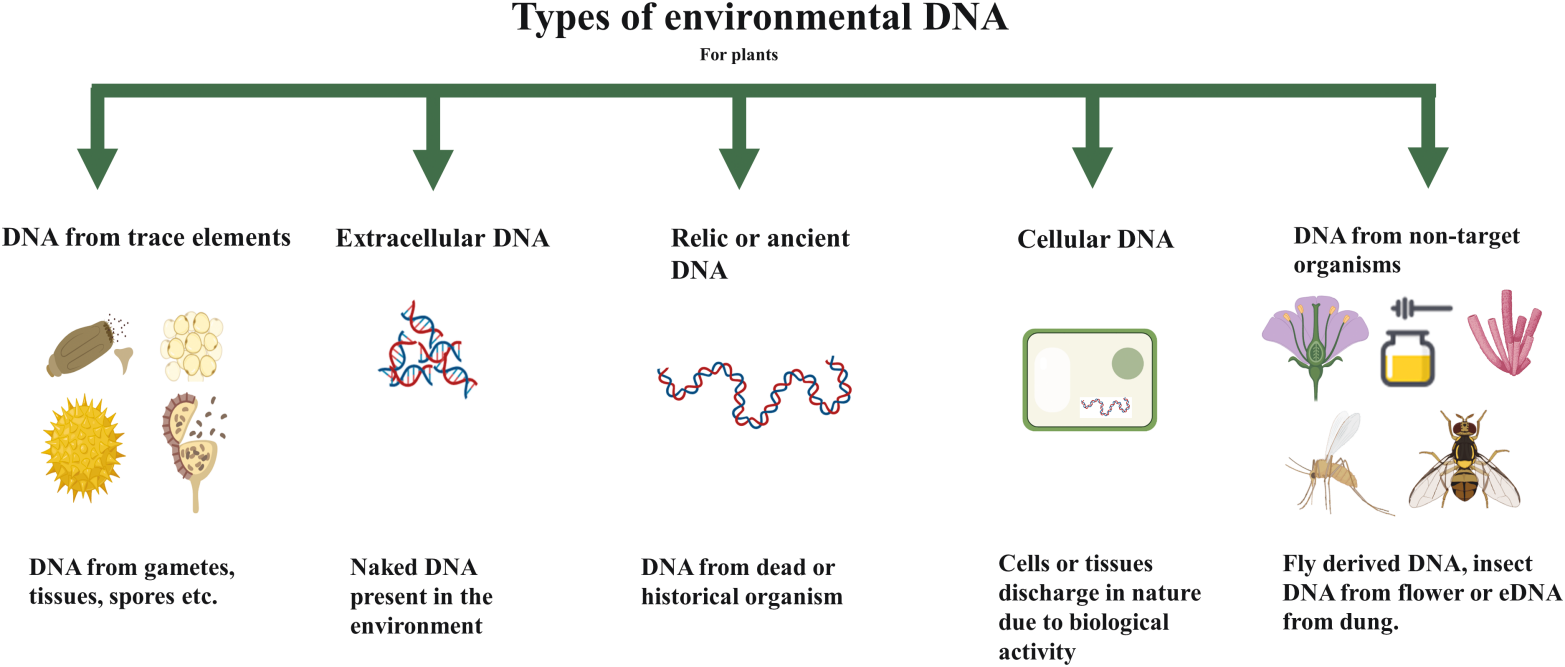
Different types of (plant) eDNA that can be collected and extracted from the environment.

Sampling methods and molecular techniques using DNA-based monitoring either from direct or bulk samples, have caught the attention of ecologists and conservation managers and have been critically evaluated in several recent reviews (Krishnamurthy and Francis 2012; Taylor and Harris 2012; Sheth and Thaker 2017; DeSalle and Goldstein 2019). The implementation of DNA barcoding (focusing on single species) and metabarcoding (barcoding coupled with high-throughput sequencing methods to detect multiple species or whole communities) in biodiversity monitoring has proved to be effective in term of detecting rare (Hosein *et al.* 2017), endangered (Lee *et al.* 2016), cryptic and invasive species (Liu *et al.* 2011; Xu *et al.* 2018), understanding community composition (Matesanz *et al.* 2019), plant–animal interactions (e.g. DNA from honey samples, diet analysis) (Pornon *et al.* 2017) and reconstructing past flora (Jørgensen *et al.* 2012; Alsos *et al.* 2018). DNA-based methods provide powerful tools for quick identification and discrimination of taxa. Furthermore, implementation of eDNA-based methods, where the collection and detection of species through DNA from air, water and soil represents a novel non-destructive approach that could revolutionize species monitoring programs (Minamoto *et al.* 2012; Miya *et al.* 2015; Deiner *et al.* 2017; Yamamoto *et al.* 2017; Cristescu and Hebert 2018; Taberlet *et al.* 2018; Ruppert *et al.* 2019; Calderón-Sanou *et al.* 2020; Banerjee *et al.* 2021). Environmental DNA is shed by organisms into their surroundings and thus lends itself to easy collection procedures. Indeed, these molecules represent remnant signatures of species, and are not only restricted to cellular DNA or extra-organismal DNA (e.g. epidermal cells, pollens, spores and other traces) but also include naked DNA (extracellular DNA) (Fig. 1) (Pawlowski *et al.* 2020b; Rodriguez-Ezpeleta *et al.* 2021).

Research employing such non-destructive eDNA-based methods in both aquatic (freshwater and marine systems) and terrestrial environments (soil and air) has provided valuable findings (Minamoto *et al.* 2012; Deiner *et al.* 2016; Berry *et al.* 2019; Ritter *et al.* 2020; Valentin *et al.* 2020). In recent decades, eDNA-based methods have been successfully employed to understand many critical concepts of ecology (e.g. habitat preference, migration, species interaction; Wu *et al.* 2019), including the detection and monitoring of focal or rare organisms where the collection of samples is critical for conservation initiatives (Stewart *et al.* 2017). The early detection of invasive species at low density (Muha *et al.* 2019), or entire communities from virgin areas (Ritter *et al.* 2020) has also been carried out for numerous taxa. But while eDNA-based methods have been successfully used for detecting a diversity of taxa, from microorganisms (Abdelfattah *et al.* 2018) to macro-organisms (Deiner *et al.* 2021), less research has focused on the development of eDNA-based methods in higher plants.

The relative paucity of eDNA applications using plants may, in part, be reflective of their (apparent) ease in traditional sampling methods, where the focal taxa are static and also potentially because of their less charismatic standing for conservation awareness in comparison to their animal counterparts (Clucas *et al.* 2008). But cross-taxon congruence between plants and animal groups is known across monitored sites and biodiversity metrics (e.g. Radford and Odé 2009), suggesting a clear and urgent need to not only identify plant conservation priorities but also increase plant-specific monitoring on a systematic and global scale for maximum impact on environmental decision-making. Here, we argue that eDNA methods could spearhead plant monitoring programs, filling up large knowledge gaps in plant biodiversity data; particularly for species of urgent conservation needs.

The slower methodological development of eDNA analysis for plants may reflect the many hurdles associated with using DNA methods for plant taxa in general (e.g. incomplete DNA reference libraries and development of universal primers) (Kress 2017). In fact, the implementation of DNA-based tools for plant species identification was initially questioned due to the shortfall of a ‘universal’ barcode. However, barcoding regions *rbcL*, *trnH–psbA*, *matK* (on the chloroplast genome) and ITS within the nucleus have now been identified and validated for such uses, making barcoding and metabarcoding options a reality (Kress 2017).

**Figure 2.**
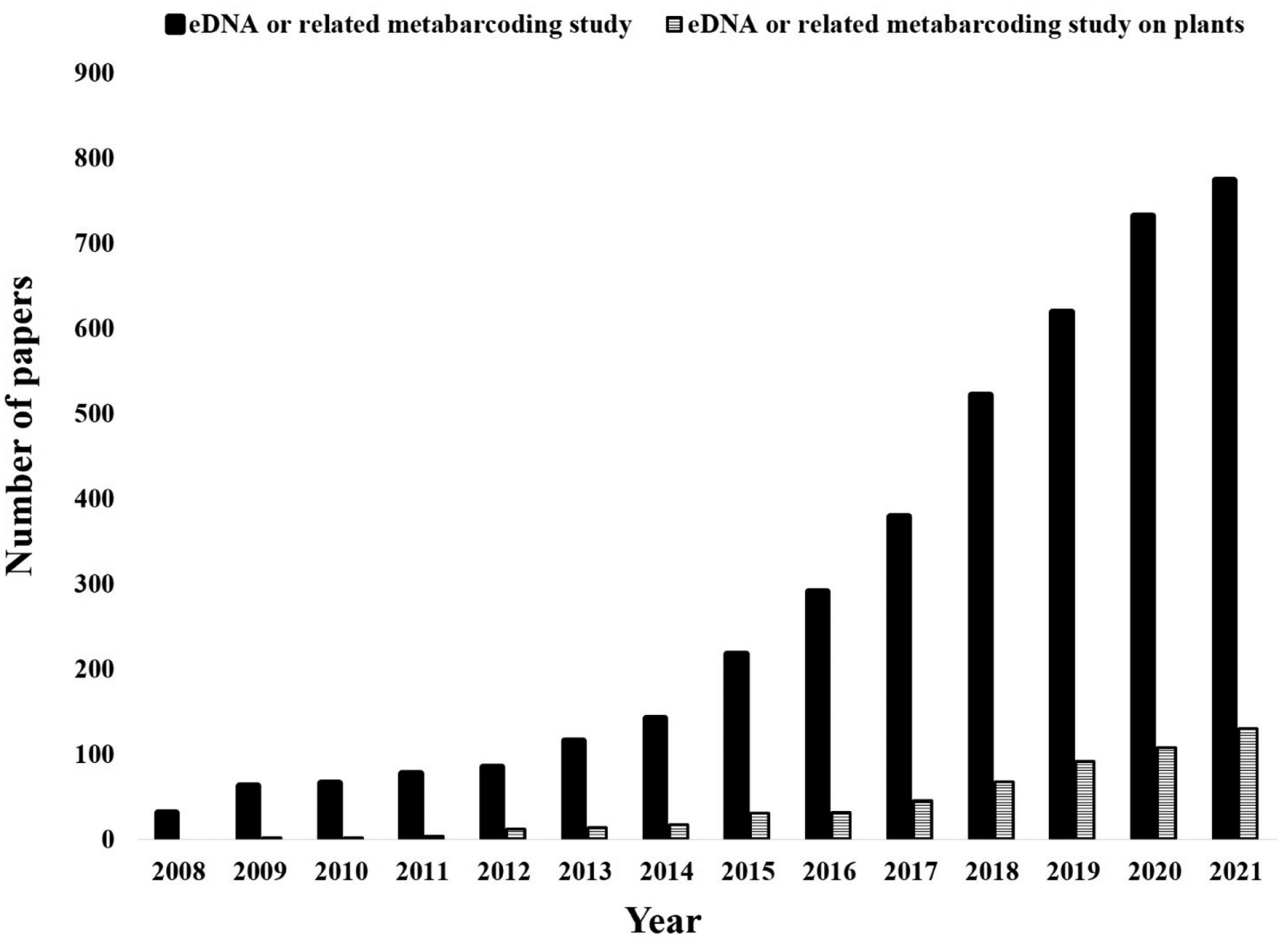
Cumulative total number of eDNA or related metabarcoding studies (solid bars) and those studies focusing specifically on plants (hashed bars). Data collected from 2008- September 2021 (06/09/22) from PUBMED.

In order to systematically review the literature, comparing studies that use eDNA for plant biomonitoring to all other eDNA studies performed to date, we searched the online database PubMed with the criteria ‘(((environmental DNA[Title/Abstract]) OR (eDNA[Title/Abstract])) OR (metabarcoding[Title/Abstract]))’ for all eDNA (e.g. barcoding or otherwise) or related metabarcoding studies, including those focused on animals or microscopic taxa. We then searched the literature using the terms ‘(((environmental DNA[Title/Abstract]) OR (eDNA[Title/Abstract])) OR (metabarcoding[Title/Abstract])) AND (plants[Title/Abstract])’ for studies specifically targeting plants, including diet (faecal) and pollinator (e.g. pollen, honey) analysis, across all plant taxa (Fig. 2). Subsequently, we then refined our search by selecting only those studies dealing with eDNA-based methods (focused on air, water, soil excluding ancient eDNA samples) and on vascular plants (pteridophytes, gymnosperms and angiosperms) (Table 1; **see**Supporting Information—**Data 1**). The endeavour was made to draw the attention of practitioners and scientists who may otherwise be unfamiliar with the achievements of the eDNA-based methods and its application in plant ecology and conservation, specifically highlighting case studies in vascular plants.

**Table 1.** Vascular plant eDNA-based monitoring studies focused on air, water and soil environments between 2008 and 2021.

eDNA target	Environment	Plant taxon	Country	Reference
Species-specific	Aquatic	*Egeria densa*	Japan, USA	(Fujiwara *et al.* 2016; Matsuhashi *et al.* 2016; Chase *et al.* 2020; Doi *et al.* 2021a; Miyazono *et al.* 2021)
		*Elodea canadensis*	USA	(Gantz *et al.* 2018; Anglès d’Auriac *et al.* 2019)
		*Hydrilla verticillata*	Japan, USA	(Matsuhashi *et al.* 2016; Gantz *et al.* 2018)
		*Potamogeton crispus*, *Stuckenia pectinata*, *P. foliosus, S. filiformis* and *Zannichellia palustris*	USA	(Kuzmina *et al.* 2018)
	Terrestrial (soil)	*Sapria himalayana*	Thailand	(Osathanunkul 2019)
Community	Aquatic	Angiosperm	Canada	(Coghlan *et al.* 2021)
			China	(Ji *et al.* 2021b)
		Podostemaceae	Japan	(Tsukamoto *et al.* 2021)
	Terrestrial (air)	Angiosperm	The Netherlands	(Kraaijeveld *et al.* 2015)
			Finland	(Korpelainen and Pietilainen 2017)
			Italy	(Banchi *et al.* 2020b)
			USA	(Johnson *et al.* 2019, 2021)
		Gymnosperm, angiosperm	Italy	(Leontidou *et al.* 2021)
			Japan	(Uetake *et al.* 2021)
			USA	(Lennartz *et al.* 2021)
		Poaceae (grass family)	UK	(Brennan *et al.* 2019b)
	Terrestrial (petal surface)	Angiosperm	Japan	(Ohta *et al.* 2018)
	Terrestrial (soil)	Pteridophyte, gymnosperm, angiosperm	Australia	(van der Heyde *et al.* 2020)
			Canada	(Fahner *et al.* 2016)
		Pteridophytes, angiosperm	Norway, France, French Guiana	(Yoccoz *et al.* 2012)

## Emergence of eDNA in Macro-organism Community Studies

The concept of eDNA-based species detections originally emerged from microbiological studies (Ogram *et al.* 1987). In these studies, DNA-based methods focused on extracellular DNA (which plays a crucial role in biofilm development) for monitoring of phytoplankton and bacterial communities. Here, researchers mostly targeted particulate, extracellular and dissolved DNA to detect DNA outside of the cell (Ogram *et al.* 1987; Rondon *et al.* 2000; Levy-Booth *et al.* 2007). In the early 2000s, the term ‘environmental DNA’ was introduced in microbial community analysis (Lakay *et al.* 2007), but implementation of eDNA to detect macroorganisms non-invasively and non-destructively did not come to the forefront until 2008, with the detection of aquatic invasive species (Ficetola *et al.* 2008). Later on, the methodology was updated by pioneer studies to detect rare aquatic animals (Darling and Mahon 2011; Jerde *et al.* 2011). Further, successive studies on eDNA persistence and transport (Dejean *et al.* 2011; Goldberg *et al.* 2011; Pilliod *et al.* 2013), release rates (Maruyama *et al.* 2014; Andruszkiewicz Allan *et al.* 2021), changes in concentration in relation to organismal abundance and seasonal activities were validated (Dejean *et al.* 2012; Takahara *et al.* 2012; Thomsen *et al.* 2012; Spear *et al.* 2015). The eDNA-based method thrived rapidly and became a multidisciplinary branch of science (Deiner *et al.* 2021). In fact, methodological optimization has remained a primary focus (Deiner *et al.* 2015; Miya *et al.* 2015; Banerjee *et al.* 2021; Bruce *et al.* 2021b), wherein, researchers have successfully utilized eDNA for species detection to reveal many ecological questions (Minamoto *et al.* 2012), such as organism presence/absence (Ficetola *et al.* 2008), abundance and habitat preference (Wu *et al.* 2019), detection of rare, threatened (Qu and Stewart 2019) and invasive species (Muha *et al.* 2019), monitoring whole biodiversity (Ritter *et al.* 2020; Yamamoto *et al.* 2017), study of species interactions (Banerjee *et al.* 2022), population ecology (Sigsgaard *et al.* 2020), behavioural biology (Dunn *et al.* 2017), anthropogenic effects (Zhang *et al.* 2020), ecosystem health (Fossøy *et al.* 2020) and even disease monitoring (Barnes *et al.* 2020) across numerous taxa.

For plants specifically, eDNA biomonitoring has been deployed using air (Longhi *et al.* 2009), soil (Yoccoz *et al.* 2012) as well as water (Matsuhashi *et al.* 2016) samples. Our literature review quantified a total of 4114 eDNA studies across all organisms, illustrating a precipitous increase in recent years. Out of these, only 558 (13 % of total) of all cumulative studies conducted to date have used eDNA-based methods to detect plant species or communities (species-specific or metabarcoding). Although, more studies incorporated eDNA-based biomonitoring on plant communities in 2020 and 2021, this number still remained low at approximately 15 % of all studies within those years (Fig. 2; **see****Supporting Information—Data 1**). However, these studies also include past biodiversity monitoring through sediment DNA/ancient DNA (Zobel *et al.* 2018b; Stoof-Leichsenring *et al.* 2020), other indirect sampling approaches, e.g. DNA from honey samples (Khansaritoreh *et al.* 2020), diet analysis (Bhattacharyya *et al.* 2019), species identification from herbal products (Raclariu *et al.* 2018), as well as DNA from the environmental samples (eDNA). Interestingly, present-day studies using eDNA-based methods (focused on air, water, soil) on vascular plants represent only 4 % of studies on plants, and <1 % of all eDNA or related metabarcoding studies that could demonstrate great utility for community- or ecosystem-level quantification and monitoring **[see****Supporting Information—Data 1****]**.

Of the available research that has utilized eDNA methods (air, water, soil) for plant detection and/or quantification, studies have successfully detected invasive, rare and endangered plants (Matsuhashi *et al.* 2016; Osathanunkul 2019) as well as entire communities (Banchi *et al.* 2020b) and their interactions (Banerjee *et al.* 2022). In fact, monitoring plant biodiversity with eDNA has been validated in both terrestrial (Fahner *et al.* 2016; Banchi *et al.* 2020b; Lentz *et al.* 2021) and aquatic (Kuzmina *et al.* 2018; Doi *et al.* 2021a) environments (Table 1). Indeed, greater methodological standardization, including development of specific primers for single-species detection and universal primers for community analysis (Scriver *et al.* 2015; Ortega *et al.* 2021), assay validation (Matsuhashi *et al.* 2016), building up reference databases (Banchi *et al.* 2020a) and comparison to traditional surveys (Gantz *et al.* 2018; Kuehne *et al.* 2020; Johnson *et al.* 2021), have all demonstrated efficient and effective application of eDNA collections.

## Workflow and Recent Advances in eDNA-Based Methods

**Figure 3.**
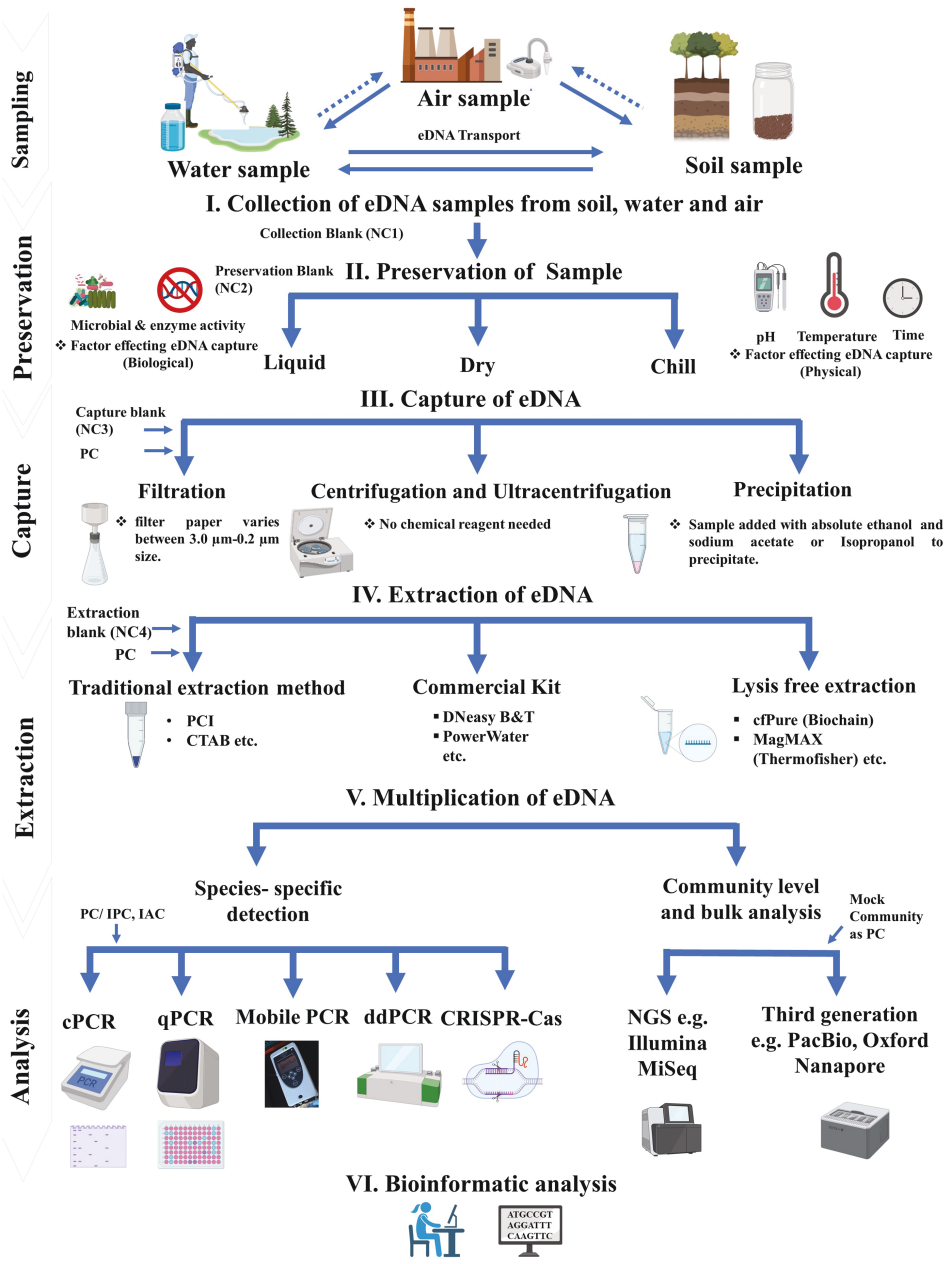
Detailed workflow of eDNA-based methods (air, water or soil). NC = negative control; PC = positive Control; IPC = internal positive control; IAC = internal amplification control; PCI = phenol/chloroform/isoamyl alcohol; CTAB = cetyl-tri-methyl-ammonium bromide; DNeasy B&T = DNeasy blood & tissue kit; PowerWater = DNeasy powerwater kit; cfPure = cell-free DNA extraction kit; MagMAX = MagMAX viral/pathogen nucleic acid isolation kit.

Traces of eDNA in general, and of plants in particular, can be detected from different environments, where the sampling approaches and extracting protocols may be modified and adapted according to the type of sample and specific aim of the study (Deiner *et al.* 2015, 2021; Bruce *et al.* 2021a). Like animals, detection of plant eDNA can be possible across large zones due to the ejection of reproductive propagules and transportation of eDNA in and between the mediums (Bell *et al.* 2016) (Fig. 3). Thus, before application of eDNA methods for plant species, methodological standardization and understanding of the habitat of target taxa are essential. Here, we do not attempt to furnish a complete guide to the methodology (see Taberlet *et al.* 2018; Tsuji *et al.* 2019; Kumar *et al.* 2020b; Bruce *et al.* 2021a; Minamoto *et al.* 2021 for further details), but summarized the total workflow in a few steps as described below.

### Sampling approaches and environmental influences (Step I)

In aquatic environments, typically a well-cleaned DNA-free bottle or one-time use sampler is suitable for collecting water from the surface (e.g. for surface plants), whereas a sampler equipped with pole/rope-like structure (e.g. Van Dorn sampler) is used for submerged water (Berry *et al.* 2019; Doi *et al.* 2021a). However, as technology is progressing to simplify sample collection and improving efficiency, replicability and sterility of water sampling, a fully integrated sampling system can also be utilized (Thomas *et al.* 2018). Furthermore, for sampling ease, mobile polymerase chain reaction (PCR) and field preparation for eDNA amplifications have also been developed to provide rapid on-site eDNA detection (Doi *et al.* 2021b), thereby rapidly scaling-up biomonitoring speed and breadth. As any strategy of eDNA sample collection may not be suitable for all organisms, an objective-based sampling strategy (e.g. sample quantity, volume, locations) should be designed prior to fieldwork (Bruce *et al.* 2021a).

In terrestrial environments, specific collection protocols for soil samples include using a sterile digger, auger or debris metal screens (Ritter *et al.* 2020), and for sediments, sterile tubes, modified plastic syringes or drilling cores. Importantly, depth of sampling may vary depending on the target taxa. For air samples, individuals can use a volumetric sampler equipped with filter paper, adhesive tape or sterile collection tubes (Brennan *et al.* 2019; Banchi *et al.* 2020b; Rowney *et al.* 2021; Tordoni *et al.* 2021). But eDNA collection is not restricted to these three habitats only and has radically advanced towards innovative point-sampling. For example, eDNA can also be sampled from non-target organisms such as insect-derived DNA to study plant diversity (Gogarten *et al.* 2020), as well as from flower surfaces to study plant–pollinators–interactions (Ushio *et al.* 2015; Ohta *et al.* 2018; Thomsen and Sigsgaard 2019). Plant–pollinator interactions and pollinator floral preferences can be also monitored by sampling pollen from the bodies of pollinators (Lucas *et al.* 2018a, b; Potter *et al.* 2019) or from honey (De Vere *et al.* 2017; Jones *et al.* 2021a); however, non-destructive monitoring approaches should be implemented if working with taxa of ecological concern.

Interpretation of species identification data with eDNA may depend upon a plant’s life history, phenotype, abundance, seasonal and reproductive activity of the taxon (Berry *et al.* 2019; Stewart 2019; Wacker *et al.* 2019; Wood *et al.* 2020). Moreover, the persistence of eDNA may depend upon the physicochemical characteristics of the environment (temperature, pH, oxygen, conductivity, moisture content, light (visible/UV) exposure, transportation and mobilization) and biotic factors (nuclease activity, microbial activity) (Stewart 2019; Wood *et al.* 2020). These factors strongly effect the final outcome; thus, understanding their role is important. Environmental DNA copy number is often related with the abundance and activity of plant species (Gantz *et al.* 2018); however, sampling seasons also influence the eDNA concentration. For example, Matsuhashi *et al.* (2019) noted eDNA concentration in aquatic plants (*Hydrilla verticillata*) significantly differed between seasons, with eDNA concentration highest during the growth period (spring to autumn) compared to dormant period (winter). Similar findings have also been reported by Doi *et al.* (2021a) in *Egeria densa* and Anglès d’Auriac *et al.* (2019) in *Elodea canadensis*. Although, the effect of these above-mentioned biotic and abiotic factors on eDNA detection has been Observed and systematically reviewed for animals (Stewart 2019), they have not fully been evaluated in plants (but see also Gantz *et al.* 2018; Matsuhashi *et al.* 2019; Doi *et al.* 2021a).

### Preservation (Step II)

Post-collection, samples are generally preserved by storing on ice or 4 °C temperature, frozen at −20 or −80 °C, dry preservation with absorbents (e.g. silica gel) (Kumar *et al.* 2020), or liquid preservation with pure preservative (e.g. ethanol, benzalkondium chloride (0.01 %)) (Jo *et al.* 2021) or lysis agents (e.g. Longmire’s buffers) (Kumar *et al.* 2020; Bruce *et al.* 2021a).

### Capture and extraction (Step III and IV)

Samples may be further processed through filtration, centrifugation, ultracentrifugation or precipitation methods to accumulate eDNA (Tsuji *et al.* 2019) but samples that are not subjected to an accumulation step can undergo direct extractions (Fig. 3). Filtration method uses fine porous membrane (e.g. 0.22 l, 0.45 l) to capture DNA; precipitation method uses ethanol and salt to precipitate DNA, whereas in centrifugation and ultracentrifugation method, DNA can be accumulated without adding any chemical (Bruce *et al.* 2021a). Filtration method are more common in use because the process larger volume of water (generally 0.5–2 μm; Tsuji *et al.* 2019); however, other methods (e.g. precipitation) can be used where collection of samples is difficult (Tsuji *et al.* 2019). Nowadays, both on-site and off-site eDNA filtration equipment are also available commercially (e.g. EnviroDNA; https://www.envirodna.com/). Moreover, implementation of these capture methods depends on volume of sample needed, which further depends on species abundance. Furthermore, there are many DNA extraction approaches and the method used can affect the quality of the resulting DNA template. It is important to test the DNA extraction method to ensure that it is suitable for the downstream DNA application (Deiner *et al.* 2017).

### Amplification and sequencing (Step V)

Target species detection focuses on a particular species (one or few) and uses species-specific primers to amplify particular targets with conventional PCR (cPCR) for ‘presence and absence’, or quantitative PCR (qPCR) for DNA copy number quantification or used for more sensitive/accurate detection when DNA molecules are scarce (Wineland *et al.* 2019). Specific primers need to be designed for the target species and validation carried out to ensure that they do not cross-amplify related taxa (Rowney *et al.* 2021). Another kind of PCR, the droplet digital PCR (ddPCR), has also demonstrated very high sensitivity (Nathan *et al.* 2014), and species detection with the CRISPR-Cas method has also been used (Williams *et al.* 2019).

On the other hand, DNA metabarcoding approaches use universal primers coupled with high-throughput sequencing to analyse many samples in parallel and can identify multiple species in each sample (Bush *et al.* 2019). Target species detection is used to monitor, quantify, as well as study the behaviour (e.g. seasonal influence) of one or few species; whilst metabarcoding is used to detect whole plant communities, study complex interactions and give equal emphasis on a large number of target taxa (Bylemans *et al.* 2019; Blackman *et al.* 2020). However, in all of the above methods, choice of markers is extremely important to detect and discriminate the target taxa. In the case of animals, universal or species-specific primers are often based on mitochondrial cytochrome c oxidase I (CO1), 12s, 16s rRNA (Hall 1999; Che *et al.* 2012), but no single barcode region has been found to be perfect in resolving all plant taxa adequately (Jones *et al.* 2021b). The low mutation rate of the mitochondrial CO1 region in higher plants makes it unsuitable, leading instead to the use of chloroplast (cpDNA) and nuclear DNA (nDNA) regions (Lee *et al.* 2016). The two core plastid DNA barcodes, cpDNA maturase K (*matK*) and ribulose-bisphosphate carboxylase (*rbcL*) gene, in combination are found to be effective for plants and especially for angiosperms (Kreft and Jetz 2007). Furthermore, cpDNA *psb–trnH* intergenic spacer and nuclear ribosomal internal transcribe spacer- ITS1 or ITS2 are also effective in species-level discrimination (Kress and Erickson 2007; Chen *et al.* 2010; Group *et al.* 2011). These barcode regions are typically used in plant barcoding and metabarcoding, but the longer length of *matK* makes its use in metabarcoding more difficult. A combination of *rbcL* and ITS2 is recommended for plant metabarcoding studies (Jones *et al.* 2021b). DNA mini-barcodes are more preferable for eDNA, due to degradation of longer fragment in environment (Hajibabaei and McKenna 2012; Little 2014), however, this may reduce taxonomic resolution.

Following amplification, most studies currently use the Illumina MiSeq platform with v3 that can provide sequence read lengths of 300–550 base pair reads. New long-read sequencing technologies (e.g. PacBio HiFi long-read sequencing) have the potential to increase sequence length, which could provide increased taxonomic resolution. Meanwhile, short-read sequencing technologies, such as Illumina NovaSeq, have the potential to increase throughput making sample processing faster and cheaper. Portable sequencing devices, like the Oxford Nanopore MinION, can allow fast analysis within the field. Thus, whole or reduced genome approaches are increasingly being used within ecological studies and have significant potential for plant monitoring.

### Bioinformatics (Step VI)

The quantity of data produced from eDNA and metabarcoding studies requires automated processes for the curation of sequences and assigning taxonomy. Various off-the-shelf as well as custom pipelines exist and the settings used within these pipelines must be thoroughly validated (Deiner *et al.* 2017). The choice of the perfect bioinformatic pipelines is important to obtain accurate results. Newly developed pipelines (Mathon *et al.* 2021) as well as existing ones (e.g. Barque, QIIME 2) can be applied according to study. Furthermore, choice between use of OTU (operational taxonomic units) and ASV (amplicon sequence variant) can also influence taxonomic assignment. OTUs overcoming PCR and sequencing error are generally clustered sequences based on a threshold similarity, whereas ASVs identify unique sequence variations also filter out, PCR and sequencing errors, providing more precise and accurate measurements of single nucleotide variations. The use of ASV is growing due to its precision, reproducibility and comprehensiveness, thus may possibly replace OTU (Callahan *et al.* 2017). Overall, the choice of these parameters will depend on the reference database, marker used and aim of study.

### Precautions

Limitations and precautions do exist with the use of eDNA methods for plants, for example, ensuring suitable primers for the questions being addressed, the requirement for standardized methodologies and the creation of suitable and complete reference libraries (Echevarria-Machado *et al.* 2005). To reduce false-positive and -negative error (including PCR inhibition) and eliminate chances of contamination during all the described steps in Fig. 3, positive controls (PC) (e.g. IPC: internal positive control, IAC: internal amplification control) and negative controls (NC) (e.g. collection blank, preservation blank, extraction blank) should be used (Jorgensen *et al.* 2020; Pawlowski *et al.* 2020a), and all possible types of error should be considered (Darling and Mahon 2011). The use of 10–50 % bleach solution followed by 75 % ethanol, DNA Away, Decon 90, DNA-exitusPlus is recommended for sterilization purposes. Furthermore, a major consideration for PCR-based approaches is how quantitative can they be considered. Quantification is affected by the combination of marker and primer used, DNA template, mixture characteristics and PCR conditions (Lamb *et al.* 2019). However, eDNA methods using metabarcoding and other amplicon-based approaches should be considered as semi-quantitative with the abundance of DNA reads treated as estimates of relative abundance (Deagle *et al.* 2019).

## eDNA in Relation to Traditional Plant Biodiversity Monitoring

### eDNA compared to traditional monitoring

#### Aquatic environment.

Environmental DNA-based monitoring has been directly compared to traditional monitoring across several studies. For example, Kuzmina and colleagues (2018) detected three rare plant species (*Potamogeton foliosus*, *Stuckenia filiformis* and *Zannichellia palustris*) that had been overlooked using traditional methods during their field visit but amplified through eDNA. Coghlan *et al.* (2021) similarly reported additional biodiversity information with eDNA-based metabarcoding, where nine alien taxa were identified, and out of them five did not have any previous records. Shackleton *et al.* (2019) compared eDNA-based metabarcoding with previous traditional monitoring data for wetland plants and found more information about endemic species. Tsukamoto *et al.* (2021) applied eDNA-based metabarcoding to detect endangered species of Podostemaceae in Japan where traditional methods were not be fruitful due to low abundance and the submerged nature of these species. In this study, Tsukamoto and colleagues (2021) detected four species that showed similarity with previous records, although they found eDNA-based monitoring to be more effective in detecting rare species than simultaneous field surveys. For information about changes in plant diversity in relation to landscape or season, Banchi *et al.* (2020b) and Uetake *et al.* (2021) have further found eDNA to be as effective as traditional methods, especially over very short periods of time. Together, these studies suggest eDNA methods for plant biomonitoring may represent a more accurate and sensitive means compared to traditional monitoring approaches.

#### Terrestrial environment.

Air eDNA includes bulk DNA (e.g. plant parts), and even naked DNA, which can be utilized in understanding the abundance, distribution and interactions of plants (Lennartz *et al.* 2021). Kraaijeveld *et al.* (2015), for example, reported that detection and identification of plants from air–eDNA metabarcoding were found to be more effective than microscopic analysis. Brennan *et al.* (2019) showed a strong relationship between air-borne pollen and the phenology of vegetation, whilst Rowney *et al.* (2021) showed a link between the abundance and composition of air-borne pollen measured using eDNA and respiratory health in humans. In fact, for plant monitoring through air samples, most traditional surveys (microscopic analysis of pollen) and even some (air) eDNA-based surveys have focused primarily on pollen samples. Interestingly, Johnson *et al.* (2019) reported that detection of plant diversity is not necessarily based on pollen nor limited to anemophilous/entomophilous species. Rather, collections may represent a broad category of biological signatures detected from air through eDNA.

Environmental DNA methods using soil have been very popular to uncover ancient DNA from sediment samples (Zobel *et al.* 2018a; Evrard *et al.* 2019; Lentz *et al.* 2021) and have even been implemented to detect large numbers of local vegetation from surface soil (Yoccoz *et al.* 2012; Fahner *et al.* 2016; Edwards *et al.* 2018). Interestingly, soil eDNA analysis helps in detecting plants with occasional appearance (e.g. where most of the body parts are present underground and only appear during flowering), where traditional surveys have historically faced difficulties in tracing them. For example, Osathanunkul (2019) developed eDNA-based methods to detect the occasionally visible endangered parasitic plant (*Sapria himalayana*) to increase its conservation success. Here, traditional surveys depended solely on flowering time but eDNA unearthed presence throughout the year. In fact, detecting a large number of taxa from soil eDNA has recently revolutionized plant biomonitoring (van der Heyde *et al.* 2020), where traditional sampling methods have been limited to above-ground visualization. Detection of plants and their interactions has also been studied with eDNA from rhizosphere samples (Montagna *et al.* 2018). Thus, eDNA has the ability to provide additional biodiversity data over traditional methods.

### eDNA coupled with traditional monitoring

Although eDNA-based methods have provided successful results in recent studies compared to traditional methods (Banerjee *et al.* 2021), both have drawbacks. Thus, combining them may reduce the chance of error for final plant biomonitoring data (Roussel *et al.* 2015; Zaiko *et al.* 2018; Banerjee *et al.* 2022). In a comparison with traditional survey (e.g. line-point interrupt survey), Johnson *et al.* (2021) found that detection rate may vary with the type of species, where as eDNA recorded more grass where as traditional survey identified more showy flowers and both of them identified equal portion of forb species. This suggests both methods have their potential limitations. In order to understand the combined effects of eDNA-based methods and traditional surveys, Ji *et al.* (2021) noted that eDNA revealed more plant taxa per sampling site, but the combination of both methods was found to be more useful. Matsuhashi *et al.* (2016) found the equal effectiveness of eDNA-based methods and visual observation in submerged aquatic plant (*H. verticillata*); however, eDNA detection was more frequent. In another aquatic invasive plant *E. densa*, eDNA was also found to be equally effective or more beneficial than traditional surveys (Fujiwara *et al.* 2016; Gantz *et al.* 2018; Chase *et al.* 2020; Doi *et al.* 2021a; Miyazono *et al.* 2021).

However, it is evident that in its early stage of implementation, collecting eDNA for plant biomonitoring is fruitful and impressive, although the presence of potential limitations needs to be considered for its further progress, such as (i) little understanding about ecology and interactions of eDNA, (ii) degradation of eDNA in environment and false-positive and -negative concerns, (iii) improvements in quantification, (iv) lack of standardized protocols, especially for plants (but see Minamoto *et al.* 2021) and practitioners adaption, (v) urgent need of reference database and group-specific primers, (vi) improvements to bioinformatics pipelines, and (vii) availability of high-through-put instrument. (Zaiko *et al.* 2018; Harper *et al.* 2019; Banerjee *et al.* 2022).

## Conclusions and Future Perspectives

Environmental DNA methods have proven to be highly successful for surveying species, populations, communities and monitoring overall biodiversity. Despite eDNA’s potential valuable role in plant biomonitoring however, many aspects to date remain unexplored. For example, we are currently experiencing worldwide degradation of forests, particularly in the tropics (40–50 % loss in forest cover; Barlow *et al.* 2016; Corlett 2016; Giam 2017; Roe 2019). We thus are in dire need of fast and effective monitoring methods, especially for these highly biodiverse regions. However, our search detected most studies incorporating eDNA methods do not occur in the tropics where species extinction is rapidly accelerating. What’s more, while eDNA metabarcoding in animals has now specific focus on particular taxonomic groups (e.g. fish, bird, insect) more focused conservation initiatives are required for particular plant groups, e.g. bryophytes, pteridophytes (but see also Brennan *et al.* 2019; Tsukamoto *et al.* 2021; Table 1). In fact, it is worthwhile to note that our literature search revealed no scientific publications pertaining to eDNA-based monitoring involving bryophytes, which happen to be the second largest plant group, next only to flowering plants. The bryophytes are often ‘pioneer species’ and have significant roles in ecosystem functioning such as soil development, nutrient cycling, hydrology and carbon budgets (O’Neill 2000; DeLucia *et al.* 2003). Furthermore, pteridophytes and gymnosperms are also equally important plant taxa that need urgent monitoring and management. The importance of these groups therefore cannot be underestimated and this calls for immediate attention. However, as biomonitoring technology keeps updating and procedures optimized, eDNA-based approaches are likely to become an extremely versatile and an essential method for plant science, despite some limitations. Biomonitoring based on eDNA will allow researchers to understand the molecular basis of plant ecological functioning, such as (i) distribution, (ii) abundance, (iii) coexistence, (iv) interactions and (v) coevolution. Recent development of environmental RNA (eRNA) and potentially in future, environmental protein (eProtein) may further lead to the molecular basis of many biological questions (e.g. health of an organism, stress response, gene expression) (Marshall *et al.* 2021; Yates *et al.* 2021). Still, elimination of potential obstacles (e.g. reference database, barcode gap) and the development of user-friendly interfaces (e.g. standardize methodology, proper bioinformatic pipelines) would contribute to improving the wide-spread implementation of these methods for plant biodiversity monitoring and conservation implementation. Sampling methodology is rapidly developing but it still may be important at this stage to couple traditional and molecular methods together as we have noticed the increase of species detection rate when both methods are employed (Ji *et al.* 2021). The latter method would provide a (i) cost-effective, (ii) accurate, (iii) versatile, (iv) safe and perhaps most importantly (v) non-destructive (Berry *et al.* 2019) approach. In this way, the scientific community could reach a more comprehensive plant monitoring program for a variety of taxa and environments, allowing scientists, managers and policymakers to provide a global framework for actionable plant biodiversity conservation.

## Supplementary Material

plac031_suppl_Supplementary_Data

## Data Availability

Not applicable.

## Notes

**Evolution & Diversity. Chief Editor:** Jeremy Beaulieu
